# Integrative Dissection of Lignin Composition in Tartary Buckwheat Seed Hulls for Enhanced Dehulling Efficiency

**DOI:** 10.1002/advs.202400916

**Published:** 2024-03-23

**Authors:** Wenqi Yang, Haiyang Duan, Ke Yu, Siyu Hou, Yifan Kang, Xiao Wang, Jiongyu Hao, Longlong Liu, Yin Zhang, Laifu Luo, Yunjun Zhao, Junli Zhang, Chen Lan, Nan Wang, Xuehai Zhang, Jihua Tang, Qiao Zhao, Zhaoxia Sun, Xuebin Zhang

**Affiliations:** ^1^ State Key Laboratory of Crop Stress Adaptation and Improvement Henan Joint International Laboratory for Crop Multi‐Omics Research School of Life Sciences Henan University Kaifeng 475004 China; ^2^ National Key Laboratory of Wheat and Maize Crop Science College of Agronomy Henan Agricultural University Zhengzhou 450002 China; ^3^ College of Agriculture Shanxi Agricultural University Taigu 030801 China; ^4^ Houji Lab of Shanxi Province Taiyuan 030031 China; ^5^ Center for Agricultural Genetic Resources Research Shanxi Agricultural University Taiyuan 030031 China; ^6^ Key Laboratory of Plant Carbon Capture and CAS Center for Excellence in Molecular Plant Sciences Chinese Academy of Sciences Shanghai 200032 China; ^7^ Shenzhen Key Laboratory of Synthetic Genomics Guangdong Provincial Key Laboratory of Synthetic Genomics Key Laboratory of Quantitative Synthetic Biology Shenzhen Institute of Synthetic Biology Shenzhen Institute of Advanced Technology Chinese Academy of Sciences Shenzhen 518055 China

**Keywords:** COMT, GWAS, lignin, seed hull, tartary buckwheat

## Abstract

The rigid hull encasing Tartary buckwheat seeds necessitates a laborious dehulling process before flour milling, resulting in considerable nutrient loss. Investigation of lignin composition is pivotal in understanding the structural properties of tartary buckwheat seeds hulls, as lignin is key determinant of rigidity in plant cell walls, thus directly impacting the dehulling process. Here, the lignin composition of seed hulls from 274 Tartary buckwheat accessions is analyzed, unveiling a unique lignin chemotype primarily consisting of G lignin, a common feature in gymnosperms. Furthermore, the hardness of the seed hull showed a strong negative correlation with the S lignin content. Genome‐wide detection of selective sweeps uncovered that genes governing the biosynthesis of S lignin, specifically two caffeic acid O‐methyltransferases (COMTs) and one ferulate 5‐hydroxylases, are selected during domestication. This likely contributed to the increased S lignin content and decreased hardness of seed hulls from more domesticated varieties. Genome‐wide association studies identified robust associations between *FtCOMT1* and the accumulation of S lignin in seed hull. Transgenic *Arabidopsis comt1* plants expressing *FtCOMT1* successfully reinstated S lignin content, confirming its conserved function across plant species. These findings provide valuable metabolic and genetic insights for the potential redesign of Tartary buckwheat seed hulls.

## Graphical abstract

As a dicotyledonous angiosperm, Tartary buckwheat seed hull processes a unique lignin chemotype primarily consist of G lignin, commonly found in gymnosperms. The population‐level correlation analysis between lignin composition and seed hull hardness revealed that the S‐depleted lignin chemotype contribute to rigid property of Tartary buckwheat seed hulls. Current study provides valuable metabolic and genetic insights for the potential redesign of Tartary buckwheat seed hulls.

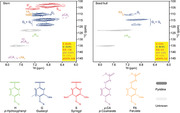

## 1 Introduction

Buckwheat is considered a functional food due to its high content of aromatic compounds, including flavonoids and phenolic acids, which are well‐documented for their antioxidant, anticancer, and anti‐inflammatory activities.^[^ ^1^, ^2^, ^3^, ^4^ ^]^ Common buckwheat (Fagopyrum esculentum Moench) and Tartary buckwheat (Fagopyrum tataricum (L.) Gaertn.) are widely domesticated and consumed around the world.^[^ ^5^ ^]^ Tartary buckwheat originated in southwest China and is currently grown on marginal lands in mountainous area and Himalayas, and in several other countries and regions, including Japan, Canada, and Europe.^[^ ^6^, ^7^ ^]^ It has height tolerance to harsh climates and acidic soils containing aluminum, which is toxic to other crops.^[^ ^8^ ^]^ Compared with common buckwheat, Tartary buckwheat, in particular, contains higher level of aromatic compounds in its seeds, such as rutin, making it of higher nutritional and medicinal value.^[^ ^9^ ^]^ However, the seeds of all Tartary buckwheat cultivars are encased in thick and rigid hulls, which are difficult to be removed during the milling process.^[^ ^10^, ^11^, ^12^ ^]^ The current dehulling process, involving boiling, results in low efficiency, broken groats, and nutrient loss, representing a significant processing bottleneck in the Tartary buckwheat industry.^[^ ^12^, ^13^ ^]^

Lignin is one of the predominant cell wall components in the seed hulls of both common and Tartary buckwheat.^[^ ^10^, ^11^, ^12^ ^]^ Lignin polymer crosslinks cell wall polysaccharides, providing strength, rigidity, and hydrophobicity.^[^ ^14^, ^15^ ^]^ Recently, by employing solid‐state nuclear magnetic resonance (ssNMR), the nanoscale interactions between lignin polymer and polysaccharides cellulose, galactoglucomannan and xylan were unveiled,^[^ ^16^, ^17^ ^]^ which could contribute the mechanical strengths of secondary cell wall. In most plant tissues analyzed to date, the lignin polymers are mainly synthesized from three monolignols, namely *p*‐coumaryl, coniferyl, and sinapyl alcohols, differing in their degree of methoxylation.^[^ ^18^ ^]^ Monolignols are synthesized in the cytosol through the phenylpropanoid pathway and are exported into the apoplastic space via diffusion driven by a concentration gradient.^[^ ^19^ ^]^ Once enter the apoplastic space, these monolignols are integrated into the growing lignin polymer through radical coupling to give rise to *p*‐hydroxyphenyl (H), guaiacyl (G), and syringyl (S) lignin units, respectively.^[^ ^14^, ^15^, ^20^ ^]^ Several cell‐wall‐bound peroxidases, laccase and mitochondrial ascorbate peroxidase participate in the oxidative lignin polymerization.^[^ ^21^, ^22^ ^]^ In addition, the Casparian strip‐located endodermal family of dirigent protein complex directs lignin polymerization on Casparian strip.^[^ ^23^ ^]^ In the polymerization process, lignin units are primarily interlinked via *β*‐O‐4 ether bonds. Interunit C–C linkages, including *β*‐*β*, *β*‐5, 5‐5, are also present in the lignin polymer.^[^ ^15^, ^24^ ^]^ C─C bonds, characterized by shorter bond lengths and higher dissociation energy than ether bonds, significantly influence the physicochemical properties of the lignin polymer.^[^ ^25^ ^]^ Different monolignols have varying numbers of methoxy groups at the aromatic ring, which could prevent the formation of interunit C–C linkages. Hence, the proportion ratio of these units determines the diversity of interunit linkages, thereby influencing the physicochemical properties of the lignin polymer.^[^ ^24^, ^25^ ^]^ For example, a higher proportion of S units would result in a lower proportion of C–C linkages, making the lignin polymer less condensed and more vulnerable to chemical or physical treatments.

Current knowledge of the lignin composition in buckwheat seed hull is limited. In this study, by investigating the lignin composition of seed hull from 274 Tartary buckwheat accessions, we unveiled a distinctive composition primarily consisting of G units (over 75%), with relatively low levels of S and H units. Correlation analysis indicated a robust negative correlation between the S unit content in the lignin polymer and the hardness of the seed hull. Genome‐wide detection of selective sweeps and association studies pinpointed that genes responsible for sinapyl alcohol biosynthesis have undergone selection during modern breeding or are associated with S unit proportion in seed hulls. Expressing *FtCOMT1*, one candidate gene that encodes a caffeic acid O‐methyltransferase, in *Arabidopsis* mutant *comt1* successfully reintroduced S lignin units into the lignin polymer. These results expanded our understanding of lignin composition and its impact on the mechanical strength of buckwheat seed hulls, providing valuable insights for further engineering desirable lignin traits to facilitate the dehulling process.

## 2 Results

### 2.1 Lignin of Tartary Buckwheat Seed Hull is Primarily Composed of G Units

**Figure 1:**
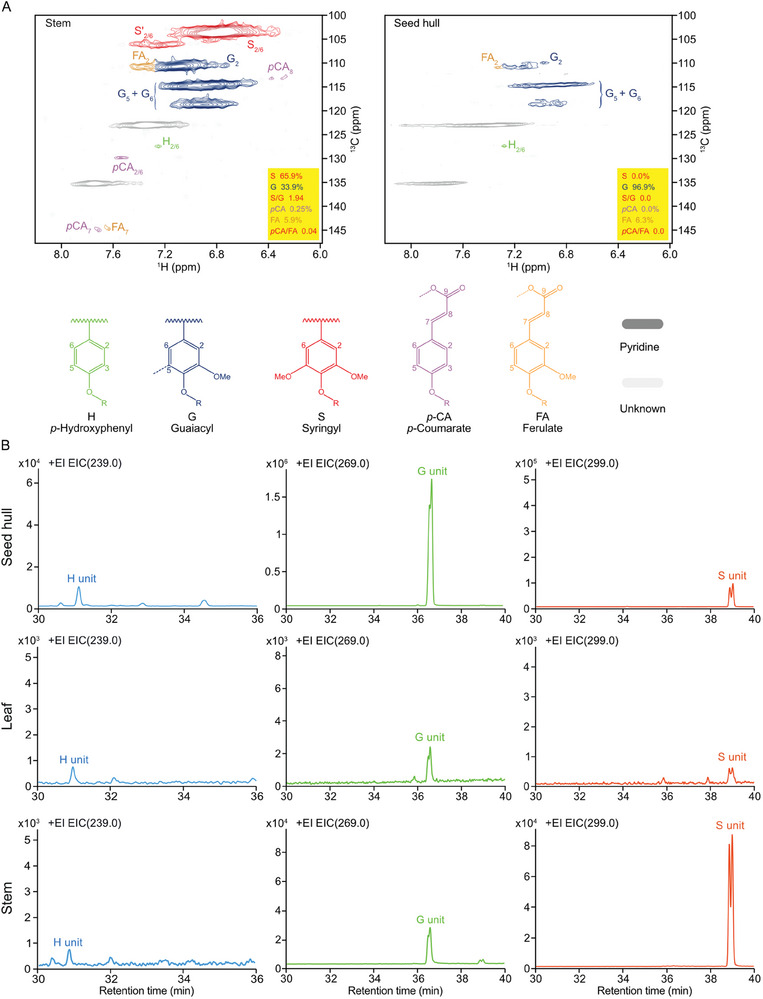
Identification of H, G, and S lignin units in Tartary buckwheat cell wall. A) 2D NMR analysis of whole cell walls from Tartary buckwheat stem and seed hull. Aromatic sub‐regions of short range ^13^C–^1^H correlation (HSQC) NMR spectra from cell wall gels of Tartary buckwheat stem and seed hull are shown. Contour coloration matches that of lignin substructures displayed in each panel. G, Guaiacyl; S, Syringyl; H, *p*‐Hydroxyphenyl; X1, Cinnamyl alcohol; FA, Ferulate; *p*CA, *p*‐Coumarate. B) Selected ion chromatograms of three lignin monomers in the thioacidolysis‐released products of indicated Tartary buckwheat cell wall from GC‐MS.

**Figure 2:**
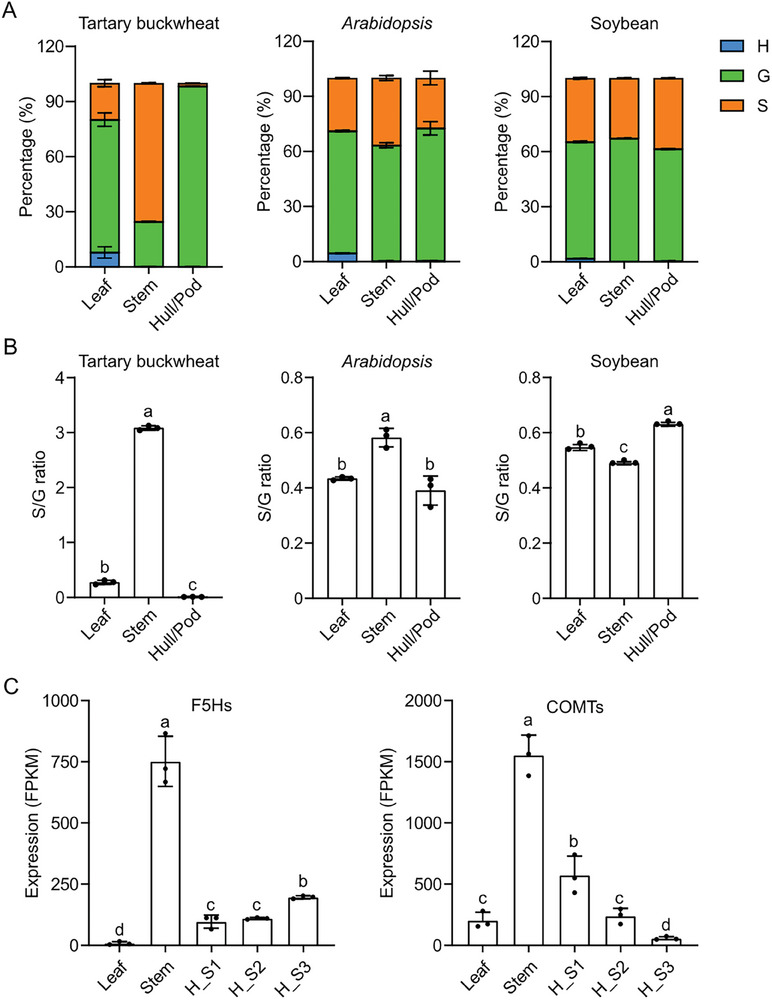
S‐depleted lignin chemotype is unique to seed hulls of Tartary buckwheat. A) Lignin composition of cell wall of indicated tissues from Tartary buckwheat, *Arabidopsis*, and soybean. Error bars represent SD. B) S/G ratio of cell wall of indicated tissues from Tartary buckwheat, *Arabidopsis*, and soybean. One‐way ANOVA followed by Tukey's honestly significant difference test was used for statistical analysis (n = 3, *p* < 0.05). All data points were plotted to show the variation of data. Error bars represent SD. Letters indicate significant differences. C) The cumulative expression of putative *F5Hs* and *COMTs* in indicated tissues. One‐way ANOVA followed by Tukey's honestly significant difference test was used for statistical analysis (n = 3, *p* < 0.05). All data points were plotted to show the variation of data. Error bars represent SD. Letters indicate significant differences.

In most dicotyledons, lignin polymer consists of G and S units, and traces of H units can also be detected in dicotyledonous angiosperm.^[^ ^18^ ^]^ The exceptional toughness of Tartary buckwheat seed hull makes us wonder whether it possesses a lignin composition that is different from other tissues or other dicots. Through solution‐state nuclear magnetic resonance (Solution NMR) spectroscopy, we analyzed the lignin composition of highly lignified stem tissue and seed hull of a Tartary buckwheat cultivar (Heifeng No. 1). For simplicity, we define the total lignin units as the sum of the H, G, and S units. The stem tissue exhibited a regular lignin composition, in which S units represent 65.9% of total lignin units, followed by 33.9% of G units and trace amount of H units (**Figure** **1**A). Interestingly, G units dominates the total lignin units (96.9%) in the seed hull, followed by 3.1% of H units, while no S units were detected (Figure 1A). We then quantified the abundance of each lignin unit in leaf, stem and see hull of this Tartary buckwheat cultivar through thioacidolysis coupled with gas chromatography‐mass spectrometry (GC‐MS). Characteristic fragment ions of H, G, and S units, that are identical to synthesized standards,^[^ ^26^ ^]^ were detected in the thioacidolysis‐released products of all tested tissues (Figure 1B, Figure S1, Supporting Information). In leaf, G units represent 72.2% of the lignin units released by thioacidolysis, followed by 19.8% of S units (**Figure** **2**A). In stem, S units represent 75.3% of the lignin units, followed by 24.5% of G unit (Figure 2A). In consistent with the solution NMR results, lignin of seed hull was almost entirely composed of G units (98.2%), with trace amount of H (0.1%) and S (1.7%) units (Figure 2A). Significant differences of the ratio between S and G units (S/G ratio) were observed among tissues of Tartary buckwheat, with the minimum of 0.016 in seed hull and the maximum of 3.07 in stem (Figure 2B). Together, these results demonstrate that G units are dominantly present, and S units are depleted, in the lignin polymer of Tartary buckwheat seed hull.

### 2.2 S‐Depleted Lignin Chemotype is Unique to Seed Hulls of Tartary Buckwheat

The extremely low level of S units in Tartary buckwheat seed hull is surprising, because G‐dominant lignin chemotype is commonly found in gymnosperms.^[^ ^18^ ^]^ This S‐depleted lignin chemotype in seed hull prompted us to investigate whether it is a common feature across different plant species. Seed hulls are specialized structure developed from ovary wall to protect the inner grains. Therefore, we analyzed the lignin composition of the seed pods of *Arabidopsis* (Col‐0 ecotype) and soybean (W82 ecotype), two well‐studied model dicotyledons, through thioacidolysis coupled with GC‐MS, as seed pods are as well developed from ovary wall. For comparison of lignin composition in different tissues, we also took the leaf and stem tissues from *Arabidopsis* and soybean for analysis. The results showed that G and S units together account for the majority of lignin units detected in all tested tissues from either *Arabidopsis* or soybean (Figure 2A). Unlike the large variance exhibited by the proportion of each lignin unit in different tissues of Tartary buckwheat, the S/G ratio remains at a comparable level (around 0.4–0.6) among different tissues in *Arabidopsis* and soybean (Figure 2B). Collectively, these results suggest that the S‐depleted lignin chemotype in seed hull is unique to Tartary buckwheat.

To explore the genetic drivers influencing the variation of S lignin unit proportion in different tissues, we performed transcriptome analysis of the leaf, stem, and seed hulls (seed hulls were harvested at three stages), and examined the expression of genes that are specialized for the biosynthesis of sinapyl alcohol. Coniferaldehyde is the branching point for the biosynthesis of coniferyl alcohol and sinapyl alcohol.^[^ ^14^, ^27^ ^]^ Ferulate 5‐hydroxylases (F5H) hydroxylate coniferaldehyde into 5‐hydroxy‐coniferaldehyde, and then it is methylated by caffeic acid O‐methyltransferases (COMTs) into sinapaldehyde.^[^ ^28^, ^29^ ^]^ Transcriptome analysis identified five copies of putative *F5H* and three copies of putative *COMT*, and the cumulative expressions of both *F5Hs* and *COMTs* are much higher in the stem tissue than other tissues (Figure 2C). These results might explain the higher S unit proportion found in the lignin of Tartary buckwheat stem tissues.

### 2.3 S Unit Content and S/G Ratio are Negatively Related to the Hardness of Tartary Buckwheat Seed Hull

**Figure 3:**
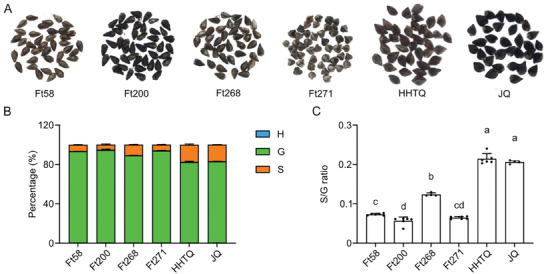
Tartary buckwheat seed hull contain less S units and lower S/G ratio than that of common buckwheat. A) Photos of seeds of four Tartary buckwheat varieties (Ft58, Ft200, Ft268, and Ft271) and two common buckwheat species (HHTQ and JQ). B) Lignin composition of seed hull cell wall of indicated varieties. Error bars represent SD. C) S/G ratio of seed hull cell wall of indicated varieties. One‐way ANOVA followed by Tukey's honestly significant difference test was used for statistical analysis (n ≥ 4, *p* < 0.05). All data points were plotted to show the variation of data. Error bars represent SD. Letters indicate significant differences.

Despite the high nutritional value of Tartary buckwheat, its rigid seed hull significantly restricts the dehulling process.^[^ ^10^, ^11^ ^]^ Because the proportion ratio of different lignin units could influence the physiochemical properties of the lignin polymer, we speculated that the S‐depleted lignin chemotype in Tartary buckwheat seed hull is related to its superior mechanical strength. It is reported that the seed hull of common buckwheat is softer than that of Tartary buckwheat.^[^ ^30^ ^]^ To examine whether the lignin polymer from common buckwheat seed hull would contain relatively more S units, we analyzed the lignin composition of seed hulls from randomly‐selected four Tartary buckwheat varieties (Ft58, Ft200, Ft268, and Ft271) and two common buckwheat varieties (HHTQ and TQ, **Figure** **3**A). As expected, the results showed that although G units account for the majority of total lignin units in the seed hulls of both buckwheat species (over 80%), the proportion of S units, as well as the S/G ratio in common buckwheat seed hull are significantly higher (Figure 3B), indicating that the low proportion of S units of Tartary buckwheat seed hull might contribute to its hardness.

**Figure 4:**
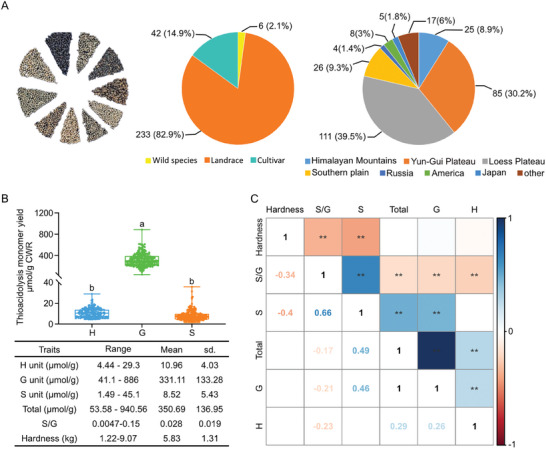
S unit content and S/G ratio are negatively related to the hardness of Tartary buckwheat seed hull. A) Population structure divided upon breeding process and geographical distribution. B) Quantification of lignin monomers in the thioacidolysis‐released products and hardness of seed hull from 274 Tartary buckwheat varieties. One‐way ANOVA followed by Tukey's honestly significant difference test was used for statistical analysis (n = 274, *p* < 0.05). All data points were plotted to show the variation of data. Error bars represent SD. Letters indicate significant differences. C) Pearson correlation among variables in (B), the value and color of square represent the magnitude and the direction of the correlation. The probability values (P‐values) less than 0.01 are marked with ^**^, indicating a strong statistical confidence.

We then performed population‐level investigation on the lignin composition (through thioacidolysis coupled with GC‐MS) and hardness of seed hulls from 274 Tartary buckwheat varieties. These 274 varieties, collected from diverse habitats across continents, exhibit high phenotypic diversity (**Figure** **4**A). Likewise, the lignin composition and hardness of seed hulls from different varieties exhibit large variation (Figure 4B). In a population scale, the overall G units account for more than 75% of total lignin units, and S units account for less than 13% (Figure 4B). Pearson's correlation analysis revealed an extremely strong correlation between the content of G units and total lignin in seed hull (Figure 4C), suggesting that variation of G unit content could largely determine the variation of total lignin unit content. Interestingly, the hardness of seed hull exhibited negative correlation with S unit content and S/G ratio, but not with other traits including G unit content (Figure 4C). In addition, the correlation between S unit content exhibited stronger correlation with S/G ratio than G unit content (Figure 4C), indicating that the content of S unit and S/G ratio might be the key drivers of seed hull hardness.

### 2.4 S Lignin Related Genes Undergone Selection During Tartary Buckwheat Breeding

**Figure 5:**
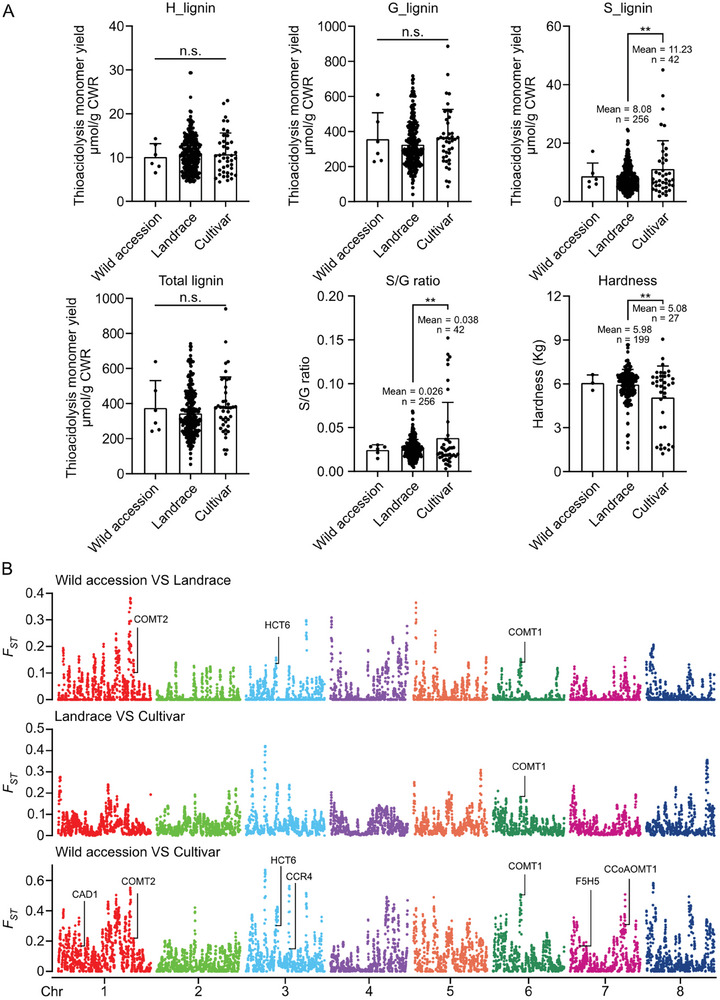
Selection Elimination of lignin related genes in geographical distribution and breeding process. A) Lignin content and hardness of seed hull from wild species, landraces and cultivars. Student's T test was used for statistical analysis. All data points were plotted to show the variation of data. Error bars represent SD. Asterisk indicate significant differences. B) Genome‐wide selection signals (*F*_ST_ value) of different Tartary buckwheat breeding stages.

Domestication and modern breeding process could significantly impact the genetic diversity of crops. As the tedious dehulling process is unfavored in Tartary buckwheat industry, we speculated that there might be a potential, if not dedicated, selection of Tartary buckwheat varieties with lower hardness of seed hull. The 274 Tartary buckwheat varieties consist of six wild accessions, 233 landraces, and 42 cultivars (Figure 4A), three groups that are representing different stages of Tartary buckwheat domestication and breeding. Hence, we examined whether differences in each lignin unit and hardness of seed hull could be detected among these groups. The results showed that the content of H and G units, as well as total lignin units, are not different among different groups (**Figure** **5**A). On the contrary, we observed a significantly higher S unit content, higher S/G ratio, and lower hardness of seed hulls in widely domesticated cultivars of Tartary buckwheat (Figure 5A), suggesting that the seed hulls of these cultivars have become softer, as a result of an altered lignin composition with more S unit proportion, during the modern breeding process.

F5H and COMT are the key enzymes for sinapyl alcohol biosynthesis.^[^ ^28^, ^29^ ^]^ The potential selection for higher S lignin units in Tartary buckwheat seed hull indicated that these genes might be selected during modern breeding process. Therefore, we performed genome‐side scanning of selection sweeps by using available genetic polymorphism data of 274 Tartary buckwheat varieties. Tartary buckwheat has a 489.3 Mb genome.^[^ ^7^ ^]^ Analysis of *F*_ST_ values identified 3551 genomic regions that are potentially selected by modern breeding process (*F*_ST_ > 0.1) (Table S3, Supporting Information). In these selective sweeps, we observed that FtPinG0009454400, which encodes a predicted O‐methyltransferase (OMT) that catalyze the methylation of various metabolites,^[^ ^31^, ^32^, ^33^ ^]^ is consistently present in a highly selected genomic region (Figure 5B).

Plant OMTs are categorized into two subgroups: caffeoyl‐CoA OMT (CCoAOMT) and COMT subgroups, according to the substrate specificity.^[^ ^33^, ^34^, ^35^ ^]^ In *Arabidopsis*, both CCoAOMT and COMT are involved in lignin biosynthesis. Because the extent of sequence identity of OMTs shows a good correlation with the structural classes of their methyl acceptor molecules,^[^ ^31^ ^]^ we performed phylogenetic analysis of FtPinG0009454400 and all OMTs in *Arabidopsis*. The result showed that FtPinG0009454400 falls within the same clade together with AtCOMT1 (Figure S2, Supporting Information). FtPinG0009454400 contains all five regions that is conserved in COMT, and shares 66% and 66.8% amino acid identity with AtCOMT1 and PtCOMT1, a COMT of poplar,^[^ ^36^ ^]^ respectively (Figure 4B), suggesting similar biochemical functions. Both AtCOMT1 and PtCOMT1 are responsible for the biosynthesis of sinapyl alcohol.^[^ ^28^ ^]^ Hence, we designated FtPinG0009454400 as *FtCOMT1*. Together, these results suggest that *FtCOMT1*, a gene likely related to S lignin unit content, had undergone selection during Tartary buckwheat breeding.

We also found that *FtCOMT2* (another putative *COMT*) was selected in the comparison between wild species and more domesticated varieties, and a putative *F5H* was selected in the comparison between wild species and cultivars (Figure 5B). Besides genes dedicated to sinapyl alcohol biosynthesis, four other genes involved in lignin biosynthesis, including *Hydroxycinnamoyl‐CoA:shikimate/quinate hydroxycinnamoyl transferase* (*HCT) 6*, *Cinnamyl alcohol dehydrogenase* (*CAD)1*, *Cinnamoyl‐CoA reductase* (*CCR*) *4* and *CCoAOMT1*, are present in the selective sweeps identified in the comparison between wild species and more domesticated varieties (Figure 5B). To further validate the identified selective sweeps, we performed Tajima's D and nucleotide diversity analysis, and focus on the genomic region containing COMTs and F5Hs, due to their specific role for S lignin biosynthesis. Both analyses suggested that these genes are located in genomic regions with reduced nucleotide diversity, at least in landrace and cultivar (Figure S3A,B, Supporting Information). We also zoomed into the genomic region containing each selected gene, and found that *F*_ST_ indicate a selective sweep at the locus. We then looked into the SNPs locating on each of the selected genes, and for two out of three genes (FtCOMT1 and FtCOMT2), we were able to find SNPs locating on the genes. Identically, the frequencies of haplotype of both FtCOMT1 and FtCOMT2 varied among three subpopulations, with landrace remaining in middle consistently (Figure S3D, Supporting Information). These results support the conclusion that these genes are selected by the tartary buckwheat breeding. Based on these results, combing with the lignin chemotype of seed hulls from Tartary buckwheat cultivars, we propose that genes related to the biosynthesis of lignin, and more specifically, S lignin units, have undergone selection during the domestication and modern breeding process of Tartary buckwheat.

### 2.5 *FtCOMT1* is Associated with S/G Ratio in Tartary Buckwheat Seed Hull

**Figure 6:**
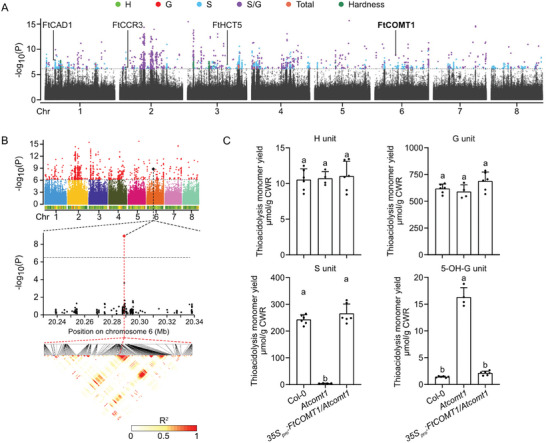
FtCOMT1 is significantly associated with the S/G ratio in Tartary buckwheat seed hulls. A) Manhattan plot of six seed hull traits analyzed by GWAS. Putative lignin biosynthesis related genes were identified within 50 kb up‐ and down‐stream of associated SNPs. B) Visualized result of GWAS for S/G ratio of Tartary buckwheat seed hull lignin. The significant‐associated SNP is highlighted in black. Sliding window represents for the 50 kb up‐ and down‐stream region surrounding the indicated significant SNP. C) Quantification of lignin monomers in the thioacidolysis‐released products of stems from indicated genotypes. One‐way ANOVA followed by Tukey's honestly significant difference test was used for statistical analysis (n ≥ 4, *p* < 0.05). All data points were plotted to show the variation of data. Error bars represent SD. Letters indicate significant differences.

To identify the genetic basis controlling the accumulation of S lignin unit in seed hull, we performed genome‐wide association studies (GWAS) by using six target traits including content of each unit, total lignin content, S/G ratio and hardness (**Figure** **6**A). In total, 3895 SNPs were identified to be associated with these traits, which defined 1078 non‐redundant QTL and 4716 candidate genes (Table S4, Supporting Information). Among the candidate genes, *FtCOMT1*, a gene that was selected during breeding of Tartary buckwheat, was found to be significantly associated S/G ratio (Figure 6A,B), suggesting strong association between *FtCOMT1* and the accumulation of S lignin units in seed hull.

COMT preferably methylate the 5‐hydroxyl group of 5‐hydroxconiferaldehyde and 5‐hydroxyconiferyl alcohol to produce the precursors of S lignin unit.^[^ ^37^, ^38^ ^]^ Plants with compromised activity of COMT show a significant decrease of S units and an increased incorporation of 5‐hydroxyguaiacyl (5‐OH‐G) lignin units.^[^ ^27^, ^28^ ^]^ To investigate whether FtCOMT1 functions similarly with AtCOMT1, we created a transgenic *Arabidopsis* line in *Atcomt1* (a COMT‐deficient mutant) background by expressing *FtCOMT1* under a 35S promoter (designated as *35S_pro_:FtCOMT1/Atcomt1*, Figure S3, Supporting Information). Analysis of the lignin composition in the highly lignified stem tissue revealed no difference in either G or H lignin content among Col‐0, *Atcomt1*, and *35S_pro_:FtCOMT1/Atcomt1*, while S lignin units are depleted and 5‐OH‐G units are highly enriched (Figure 6C), which is consistent with previous reports.^[^ ^28^, ^39^ ^]^ Heterologous expression of *FtCOMT1* in *Atcomt1* restored the content of both S unit and 5‐OH‐G unit to the wild‐type levels (Figure 6C), suggesting a conserved function of COMT1 in both Tartary buckwheat and *Arabidopsis*. Taken together, these results suggest that FtCOMT1, a dedicated enzyme related to S lignin units that had undergone selection during domestication and breeding process of Tartary buckwheat, controls the S/G ratio in the lignin polymer of seed hull, representing a promising breeding target for enhancing dehulling efficiency in Tartary buckwheat industry.

## 3 Discussion

Plants have evolved highly differentiated structures to execute desired functions. Secondary cell wall provides mechanical strength for plant bodies. One of the key cell wall components, lignin, is a complex phenolic polymer that varies in content and composition among different plant tissues and affects the secondary wall mechanical properties. Previous studies have shown a correlation between lignin absorbance and tensile stiffness in poplar trees (Özparpucu et al., 2017) and the influence of lignin biosynthesis on cell wall characteristics using specific mutants or small‐scale varieties.^[^ ^15^, ^24^ ^]^ However, the impact of lignin composition on cell wall mechanical properties is still poorly understood. In this study, we analyzed a large population of 274 Tartary buckwheat varieties with diverse lignin composition and seed hull hardness. We found that Tartary buckwheat seed hull processes a unique lignin chemotype that primarily consist of G lignin, commonly found in gymnosperms (Figure 2). The lower S unit content makes harder seed hulls in Tartary buckwheat compared with common buckwheat (Figure 3), as indicated by the negative correlation between the hardness and the S lignin content or S/G ratio of Tartary buckwheat seed hull (Figure 3). These experimental evidences collectively indicate a close relationship between lignin composition and cell wall mechanical properties.

During the polymerization process, lignin units are inter‐linked not only via *β*‐O‐4, but also through C–C linkage, including *β*‐*β*, *β*‐5, and 5‐5 bonds.^[^ ^24^ ^]^ C─C bonds are, characterized by shorter bond lengths and higher dissociation energy than ether bonds.^[^ ^25^ ^]^ Therefore, the presence of more C─C bonds leads to, the denser and more resistant lignin polymers. Recently, we identified that maize sporopollenin, which stand for the most indestructible biopolymer, primarily consists of H unit,^[^ ^40^ ^]^ This unique lignin composition not only protect the pollens against heat waves and excess UV radiation during the growth season,^[^ ^40^ ^]^ but also likely provides the sporopollenin with superior durability that could makes it persist in the sediments for over millions of years.^[^ ^40^, ^41^ ^]^ This indicated that, denser lignin confers more rigid property for cell wall. Compared with coniferyl alcohol, sinapyl alcohol forms less C–C linkage, because the methoxy groups at the aromatic ring can prevent the formation of *β*‐5 and 5‐5 C–C linkages. Therefore, higher S unit content or S/G ratio likely results in looser structure that contributes to the reduction of seed hull hardness.

Breeding of tartaty buckwheat is still in its infancy and is mostly towards a higher content of nutrients such as rutin. Through genome‐wide analysis of selective sweeps, we showed that genes that are responsible for higher S lignin units are continuously selected during the domestication and breeding process of Tartary buckwheat, which likely explains the higher S/G ratio and lower hardness of seed hulls of widely domesticated cultivars. One of the selected genes, *FtCOMT1*, is also demonstrated to be strongly associated with the S/G ration of seed hull. COMT is an important enzyme in the phenylpropanoid metabolic pathway that synchronously evolved from N‐acetylserotonin methyltransferase (ASMT) with appearance of land plant by gene duplication and subsequent divergence.^[^ ^42^ ^]^ Newly emergent COMTs are more conserved the amino acid level but possess more open conformation compared to ASMT, getting a new function on catalyzing the biosynthesis of monolignols.^[^ ^42^ ^]^ This development directly contributed to plant colonization of land and the dominance of vascular plants.^[^ ^42^ ^]^ The crucial function of COMTs in influencing S unit content (and S/G ratio) in lignin polymer makes it a promising target for engineering lignin composition of transgenic plants, to improve forage digestibility, pulping efficiency, or utility in biofuel production.^[^ ^24^, ^43^ ^]^ Therefore, higher S/G ratio and lower hardness of Tartary buckwheat seed hull would also bring extra benefits. The high S/G ratio of lignin in Tartary buckwheat stem indicate that the synthesis pathway of S unit is active in this plant. Considering the expression pattern of *COMTs* and *F5Hs*, a pathway that regulates the differential composition of S units in different tissues by controlling the tissue‐specific expression of *COMTs* and *F5Hs* might exist in Tartary buckwheat. Exploring and dissecting this pathway is of benefit in redesign of Tartary buckwheat seed hulls.

Lodging and seed shattering are two other problems that affect the yield and harvesting of Tartary buckwheat. Tartary buckwheat plants have tall and brittle hollow stems, which make them prone to lodging, bending, and pest attack. Lodging resistance affects yield and mechanical harvesting significantly (Hagiwara et al., 1999). Almost all buckwheat varieties have indeterminate inflorescence, which results in a long flowering period and a broad grain ripening period. Seeds of different maturity stages are present at the same time during the ripening period (Funatsuki et al., 2000). This makes it challenging to determine the optimum harvesting time to avoid the shedding of early‐mature seeds and minimize the yield loss (Oba et al., 1998). Seed shattering problem reduces the yield of buckwheat by about 40–50% when harvested by machine compared to hand harvesting (Radics and Mikóházi, 2010). The shattering problem is more severe in Tartary buckwheat, because its pedicel has smaller diameter, breaking bending strength, and breaking tensile strength than those of common buckwheat (Oba et al., 1998). Therefore, enhancing the physical strength of pedicel is one of the effective ways to reduce the yield loss during harvesting. Further exploration of the lignin composition of specific tissues and the regulatory mechanism in Tartary buckwheat has great potential for improving the variety and increasing the yield. In summary, this work provides a theoretical basis for further improving the mechanical properties and dehulling efficiency of Tartary buckwheat. Moreover, the genetic insights on the lignin composition of tartaty buckwheat offered by this work could largely facilitate the targeted breeding process.

## 4 Experimental Section

### Plant Materials and Growth Conditions

All buckwheat materials were grown during the cropping season (March to September of 2018 and 2019) on the experimental farm of the College of Agronomy, Shanxi Agricultural University, Taigu, Shanxi, China (37°12′N, 112°28′E). For *Arabidopsis*, Col‐0 ecotype, and T‐DNA insertion mutants *Atcomt1* (Salk_135290) were preserved in the lab. The detailed growth condition was the same as described previously.^[^ ^44^ ^]^ For soybean, William 82 ecotype was preserved in the lab, and planted in greenhouse with a temperature setting as 25 °C.

A total of 274 Tartary buckwheat accessions, including 245 varieties from Himalayan region (10 germplasms), Yun‐Gui Plateau region (89 germplasms), Loess Plateau region (118 germplasms), and Southern Plain region (26 germplasms) of China, and 29 varieties from United States, Nepal, Bhutan, Japan, and Russia, were used for correlation analysis and GWAS in this study (Table S1, Supporting Information). The hulls of mature seeds were collected for lignin composition and hardness analysis.

### Preparation of Cell Wall Residues

All tissues were collected after buckwheat seeds were fully matured. The preparation of cell wall residues and thioacidolysis was performed as described previously.^[^ ^40^ ^]^ Briefly, the fine‐milled tissue powders were extracted with 70% ethanol at 65 °C for three times each for 1 h. The residues were extracted with chloroform/methanol (1:1, *v*/*v*) and then washed by acetone, and each for three times. The residues were dried overnight and de‐starched by amylase and pullulanase. After briefly washing with water and acetone, the residues were dried overnight, resulting in de‐starched extractive‐free cell wall residues (CWRs). The CWRs were enzymatically hydrolyzed at 37 °C for 2 days using a mixture of Macerozym R10 and Cellulase Onozuka R10 (Yakult, Nishinomiya, Japan) (1% of each in 0.1 m sodium acetate buffer, pH 4.5). The residues were pelleted and washed with water and acetone. The over‐night dried residues were considered as cellulolytic enzyme lignin (CEL).

### 2D NMR Spectroscopy

Transfer 60 mg ball‐milled CEL into a 5 mm NMR tube. Add 500 µL of DMSO‐d6/pyridine‐d5 (4:1; *v*/*v*) into the NMR tube, and treated with ultrasonic bath for 1–3 h until the sample becomes apparently homogeneous. NMR spectra were acquired on Agilent 600 MHz spectrometer equipped with a 5 mm 1H‐(13C/15N) 13C Enh Cryo‐Probe. The parameters of adiabatic Nuclear Magnetic Resonance Spectroscopy (HSQC) NMR experiments (“gNhsqc”) were consistent with the reference.^[^ ^45^ ^]^ MestReNova 12 was used for NMR data analysis and processing. The central DMSO solvent peak was used as an internal reference (δH/δC 39.5 ppm/2.49 ppm). The typical matched Gaussian apodization in F2, squared cosine‐bell and one level of linear prediction (32 coefficients) in F1 to obtain the HSQC plots. The signals were assigned according to the previous literature.^[^ ^45^, ^46^ ^]^ For quantification analysis of lignin aromatic units, C2‐H2/C6‐H6 or C2′ ‐H2′/C6′‐H6′correlations form S2/6, S′2/6 and H2/6 and C2‐H2 correlations form G2 were integrated and the S2/6, S′2/6 and H2/6 signals were logically halved. The values of relative signal intensities in Figure 1 were expressed on 1/2(S2/6+S′2/6) + G2 + 1/2 H2/6 = 100 basis.

### Thioacidolysis of CWRs

10 mg CWRs was incubated with 1 mL reaction solution (dioxane: boron trifluoride diethyl etherate: ethanethiol = 175:5:25, *v*/*v*/*v*) at 100 °C for 4 h. Gently mix the samples every hour, and in the last hour, mix the samples every 20 min. After the reaction mixture was cooled, its pH was adjusted to 3–4 by 0.4 m NaHCO_3_ and then extracted with 1 mL dichloromethane. The dichloromethane layer was filtered through anhydrous sodium sulfate into a 2‐mL Eppendorf tube, and the solvent was evaporated. The resulting residues were re‐dissolved in 50 µL pyridine, and derivatized using 50 µL N,O‐bis(trimethylsilyl)trifluoroacetamide (BSTFA) at room temperature for 5 h. Aliquots of this solution were analyzed by GC/MS.

### Gas Chromatography–Mass Spectrometry

An Agilent 7820A gas chromatography coupled to a 5977B mass spectrometer was used for detection of lignin monomers presented the thioacidolysis lysate. Separation was achieved on an Agilent HP‐5MS column (30 mm × 0.25 mm, 0.25 µm film thickness). The following temperature gradient was used with a 2‐min solvent delay: Initial hold at 130 °C for 3 min; a 3 °C per minute ramp to a 250 °C and hold for 5 min. The flow rate was set at 1.1 mL per minute. Peaks were identified by characteristic mass spectrum ions of 239, 269, and 299 m z^−1^ for H, G and S units, respectively.^[^ ^26^ ^]^ The calculation of lignin monomer quantify is based on the equation as follows.

(1) $$C=\frac{\frac{\mathrm{AS}}{\mathrm{AIS}}\times \mathrm{WIS}\;\times K}{\mathrm{CWR}}$$

In this equation, C refers to the concentration (µmol g^−1^), A_S_ refers to the peak area of lignin unit, A_IS_ refers to the peak area of internal standard, W_IS_ refers to the amount (µmol) of the internal standard, K refers to the response factors of lignin units versus internal standard, CWR refers to the mass (g) of cell wall residues. In our protocol, 0.1 mg 4,4′‐ethylenebisphenol was added into 1 mL dichloromethane as internal standard. Thus, W_IS_ is 0.467, K of H, G, and S units are 1.24, 1.56, 1.91, respectively.^[^ ^26^ ^]^

### Hardness of Tartary Buckwheat Seed Hull

The seed hull was peeled from mature seeds. Hardness of seed hull were measured using a Grain Hardness Tester (WDGAGE, Model: GW‐1).

### RNA Sequence Analysis

Leaf, stem, and seed hulls at three developmental stages (S1: grain filling stage, S2: grain green mature stage, S3: grain mature stage) were used for RNA isolation and each stage has three replicates. The total RNA of samples was extracted according to the manual instructions in the RNeasy plant mini kit (Qiagen, Germany) kit, and then the quality was examined. Complementary DNA (cDNA) libraries were constructed by the Beijing Biomax Biotechnology Co., Ltd, then sequenced by the Illumina high‐throughput sequencing platform. Trimmomatic (version) was used to perform quality control on the raw sequence data.^[^ ^47^ ^]^ HISAT was adopted to compare the clean data to the reference genome,^[^ ^7^, ^48^ ^]^ whereas StringTie was applied to compare and assemble the reads.^[^ ^49^ ^]^ FPKM (fragments per kilobase of transcript per million fragments mapped) value was used for the quantification of gene expression.

### Genome‐Wide Detection of Selective Sweeps

Genome‐wide detection of selective sweeps was performed by using the *F*_ST_ value as an indicator. Population differentiation analyses were conducted between different subgroups by using vcftools software.^[^ ^50^ ^]^ For analysis of *F*_ST_ (Fixation index), the sliding window was set to 500 kb, and the step size was set to 60 kb. The *F*_ST_ values ranged from 0 to 1, with a higher value indicating a greater degree of differentiation and a higher level of selection for the QTL/gene(s).

### Genome‐Wide Association Analyses

The genotype dataset of 274 lines of Tartary buckwheat used in this study includes 1444061 SNPs (1.44 m SNPs) with a minimum allele frequency (MAF) of ≥ 0.05. Integrating genotype and phenotype data, the PCA+K model — a Mixed Linear Model (MLM) that corrects for both principal component analysis (PCA) and relative kinship (K) — was employed utilizing TASSEL 3.0 software for conducting a genome‐wide association study (GWAS).^[^ ^51^, ^52^ ^]^ The MLM model was represented by the equation y = X*α* + Z*β* + Wµ + e, where y represents trait values. The components include Xα (principal component, acting as a covariate) and Z*β* (SNPs or marker effects) as fixed effects, Wµ (relative kinship matrix) as the random effect, and e as the residual error.^[^ ^53^ ^]^ To avoid the occurrence of type I and type II errors (false positives and false negatives), a stringent threshold (1/1444061) was utilized to assess the presence of a significant association between the SNP and target trait. As reported in previous studies, a QTL was defined as a total interval of 100 kb, encompassing 50 kb upstream and downstream of the significant SNP.^[^ ^54^ ^]^ The confidence interval of significant QTL was compared with the Pinku1 reference genome^[^ ^7^ ^]^ (http://www.mbkbase.org/Pinku1) to identify genes potentially involved in regulating Tartary buckwheat hull. For visualization, the “CMplot” package was utilized in R to generate Manhattan plots, and used the “LDheatmap” package for LD plots.

### Phylogenetic Analysis

The protein sequences of OMT family in *Arabidopsis* (AT1G67980, AT1G67990, AT1G24735, AT4G26220, AT4G34050, AT3G62000, AT3G61990, AT4G35150, T4G35160, AT1G33030, AT3G53140, AT1G51990, AT1G21120, AT1G21130, AT1G21110, AT1G21100, AT1G76790, AT5G53810, AT5G37170, AT1G62900, AT1G63140, AT1G77530, AT1G77520) were retrieved from TAIR. The protein sequence of FtPinG0009454400 were extracted from the reference genome for Pinku1. The Maximum‐Likelihood phylogenetic tree (bootstrap value set as 1000) was constructed based on the protein sequences of these homologs by using MEGA X software.^[^ ^55^ ^]^

### Transformation of Plants

For the complementation of *Atcomt1* with FtCOMT1, the coding sequence of *FtCOMT1* was cloned into the entry vector pCR8 using NovoRec plus One step PCR Cloning Kit (NovoProtein), and then subcloned into the destination vector pMDC83 vector. The construct was transformed into *Agraobacterium tumefaciens* GV3101, and then transformed into *Arabidopsis* plants using floral dip method.^[^ ^56^ ^]^ For screening transgenic lines, positive transformants were screened using hygromycin (Roche).

### Statistical Analysis of Phenotypic Traits

Statistical analysis was performed by using GraphPad Prism 9 software. For each figure, the statistical method was specified in the figure legend. Pearson correlation among all traits were calculated R function stats::corr.test and visualized by R package corrplot (https://cran.r‐project.org/web/packages/corrplot/citation.html).

## Conflict of Interest

The authors declare no conflict of interest.

## Author Contributions

W.Y., H.D., K.Y., and S.H. contributed equally to this work. X.B.Z. and Z.X.S. conceived and supervised the project. X.B.Z., Z.X.S., W.Q.Y., H.Y.D., K.Y. and S.Y.H. designed the experiments. W.Q.Y., H.Y.D., Y.F.K., and X.W. performed most of the experiments and analyzed the data. S.Y.H., J.Y.H., and L.L.L. performed the transcriptome analysis. L.F.L. and Y.J.Z. performed NMR spectroscopy. Y.Z., J.L.Z., and C.L. assisted in experiments including sample preparation and GC‐MS. X.H.Z. and J.H.T helped in experimental design. Q.Z. and N.W. helped in manuscript writing. W.Q.Y., H.Y.D., K.Y., and S.Y.H. prepared graphs, and wrote the manuscript with input from all co‐authors. All authors read and approved the manuscript.

## Supporting information

Supporting Information

Supporting Information

## Data Availability

The data that support the findings of this study are available from the corresponding author upon reasonable request.

## References

[1] A.‐M. Boudet , Phytochemistry 2007, 68, 2722. 17643453 10.1016/j.phytochem.2007.06.012

[2] C. M. Fraser , C. Chapple , The Arabidopsis Book 2011, 9, e0152. 22303276 10.1199/tab.0152 PMC3268504

[3] J. A. Giménez‐Bastida , H. Zielinski , M. Piskula , D. Zielinska , D. Szawara‐Nowak , Mol. Nutr. Food Res. 2017, 61, 1600475. 10.1002/mnfr.201600475 PMC6599964 27709826

[4] C. Martin , Curr. Opin. Biotechnol. 2013, 24, 344. 23246231 10.1016/j.copbio.2012.11.005

[5] J. Giménez‐Bastida , M. Piskuła , H. Zieliński , Pol. J. Food Nutr. Sci. 2015, 65, 9.

[6] Y. He , K. Zhang , Y. Shi , H. Lin , X. Huang , X. Lu , Z. Wang , W. Li , X. Feng , T. Shi , Q. Chen , J. Wang , Y. Tang , M. A. Chapman , M. Germ , Z. Luthar , I. Kreft , D. Janovská , V. Meglic , S.‐H. Woo , M. Quinet , A. R. Fernie , X. Liu , M. Zhou , Genome Biol. 2024, 25, 61. 38414075 10.1186/s13059-024-03203-z PMC10898187

[7] L. Zhang , X. Li , B. Ma , Q. Gao , H. Du , Y. Han , Y. Li , Y. Cao , M. Qi , Y. Zhu , H. Lu , M. Ma , L. Liu , J. Zhou , C. Nan , Y. Qin , J. Wang , L. Cui , H. Liu , C. Liang , Z. Qiao , Mol. Plant 2017, 10, 1224. 28866080 10.1016/j.molp.2017.08.013

[8] H. Wang , R. F. Chen , T. Iwashita , R. F. Shen , J. F. Ma , New Phytol. 2015, 205, 273. 25195800 10.1111/nph.13011

[9] L. Sinkovič , D. Kokalj Sinkovič , V. Meglič , Food Chem. 2021, 365, 130459. 34216911 10.1016/j.foodchem.2021.130459

[10] W. Biel , R. Maciorowski , Ital. J. Food Sci. 2013, 25, 384.

[11] K. Dziedzic , D. Górecka , M. Kucharska , B. Przybylska , Food Res. Int. 2012, 47, 279.

[12] C. Song , C. Ma , D. Xiang , Int. J. Mol. Sci. 2019, 20, 524. 30691178 10.3390/ijms20030524 PMC6387337

[13] H. Y. Li , C. X. Wu , Q. Y. Lv , T. X. Shi , Q. J. Chen , Q. F. Chen , Bmc Plant Biol. 2020, 20, 505. 33148168 10.1186/s12870-020-02715-7 PMC7640676

[14] N. D. Bonawitz , C. Chapple , Annu. Rev. Genet. 2010, 44, 337. 20809799 10.1146/annurev-genet-102209-163508

[15] R. Vanholme , B. Demedts , K. Morreel , J. Ralph , W. Boerjan , Plant Physiol. 2010, 153, 895. 20472751 10.1104/pp.110.155119 PMC2899938

[16] A. Kirui , W. Zhao , F. Deligey , H. Yang , X. Kang , F. Mentink‐Vigier , T. Wang , Nat. Commun. 2022, 13, 538. 35087039 10.1038/s41467-022-28165-3 PMC8795156

[17] O. M. Terrett , J. J. Lyczakowski , L. Yu , D. Iuga , W. T. Franks , S. P. Brown , R. Dupree , P. Dupree , Nat. Commun. 2019, 10, 4978. 31673042 10.1038/s41467-019-12979-9 PMC6823442

[18] W. Boerjan , J. Ralph , M. Baucher , Annu. Rev. Plant Biol. 2003, 54, 519. 14503002 10.1146/annurev.arplant.54.031902.134938

[19] M. L. Perkins , M. Schuetz , F. Unda , K. T. Chen , M. B. Bally , J. A. Kulkarni , Y. Yan , J. Pico , S. D. Castellarin , S. D. Mansfield , A. L. Samuels , The Plant Cell 2022, 34, 2080. 35167693 10.1093/plcell/koac051 PMC9048961

[20] J. Ralph , K. Lundquist , G. Brunow , F. Lu , H. Kim , P. F. Schatz , J. M. Marita , R. D. Hatfield , S. A. Ralph , J. H. Christensen , W. Boerjan , Phytochem. Rev. 2004, 3, 29.

[21] J. Zhang , Y. Liu , C. Li , B. Yin , X. Liu , X. Guo , C. Zhang , D. Liu , I. Hwang , H. Li , H. Lu , Nat. Plants 2022, 8, 828. 35851622 10.1038/s41477-022-01181-3

[22] Q. Zhao , J. Nakashima , F. Chen , Y. Yin , C. Fu , J. Yun , H. Shao , X. Wang , Z.‐Y. Wang , R. A. Dixon , The Plant Cell 2013, 25, 3976. 24143805 10.1105/tpc.113.117770 PMC3877815

[23] Y.‐Q. Gao , J.‐Q. Huang , G. Reyt , T. Song , A. Love , D. Tiemessen , P.‐Y. Xue , W.‐K. Wu , M. W. George , X.‐Y. Chen , D.‐Y. Chao , G. Castrillo , D. E. Salt , Science 2023, 382, 464. 37883539 10.1126/science.adi5032

[24] J. Ralph , C. Lapierre , W. Boerjan , Curr. Opin. Biotechnol. 2019, 56, 240. 30921563 10.1016/j.copbio.2019.02.019

[25] L. Dong , L. Lin , X. Han , X. Si , X. Liu , Y. Guo , F. Lu , S. Rudic , S. F. Parker , S. Yang , Y. Wang , Chem 2019, 5, 1521.

[26] F. Yue , F. Lu , R. C. Sun , J. Ralph , J. Agric. Food Chem. 2012, 60, 922. 22191493 10.1021/jf204481x

[27] D. Guo , F. Chen , K. Inoue , J. W. Blount , R. A. Dixon , Plant Cell 2001, 13, 73. 11158530 10.1105/tpc.13.1.73 PMC102215

[28] T. Goujon , R. Sibout , B. Pollet , B. Maba , L. Nussaume , N. Bechtold , F. Lu , J. Ralph , I. Mila , Y. Barrière , C. Lapierre , L. Jouanin , Plant Mol. Biol. 2003, 51, 973. 12777055 10.1023/a:1023022825098

[29] J. M. Marita , J. Ralph , R. D. Hatfield , C. Chapple , Proc. Natl. Acad. Sci. USA 1999, 96, 12328. 10535921 10.1073/pnas.96.22.12328 PMC22916

[30] S. Q. Li , Q. H. Zhang , Crit. Rev. Food Sci. Nutr. 2001, 41, 451. 11592684 10.1080/20014091091887

[31] R. K. Ibrahim , A. Bruneau , B. Bantignies , Plant Mol. Biol. 1998, 36, 1. 9484457 10.1023/a:1005939803300

[32] K. C. Lam , R. K. Ibrahim , B. Behdad , S. Dayanandan , Genome 2007, 50, 1001. 18059546 10.1139/g07-077

[33] S. Roje , Phytochemistry 2006, 67, 1686. 16766004 10.1016/j.phytochem.2006.04.019

[34] C. P. Joshi , V. L. Chiang , Plant Mol. Biol. 1998, 37, 663. 9687070 10.1023/a:1006035210889

[35] J. Raes , A. Rohde , J. H. Christensen , Y. Van de Peer , W. Boerjan , Plant Physiol. 2003, 133, 1051. 14612585 10.1104/pp.103.026484 PMC523881

[36] B. Dumas , J. Van Doorsselaere , J. Gielen , M. Legrand , B. Fritig , M. Van Montagu , D. Inzé , Plant Physiol. 1992, 98, 796. 16668718 10.1104/pp.98.2.796 PMC1080267

[37] L. Li , J. L. Popko , T. Umezawa , V. L. Chiang , J. Biol. Chem. 2000, 275, 6537. 10692459 10.1074/jbc.275.9.6537

[38] K. Parvathi , F. Chen , D. Guo , J. W. Blount , R. A. Dixon , Plant J. 2001, 25, 193. 11169195 10.1046/j.1365-313x.2001.00956.x

[39] J. K. Weng , H. Mo , C. Chapple , Plant J. 2010, 64, 898. 21143672 10.1111/j.1365-313X.2010.04391.x

[40] W. Yang , D. Yao , H. Duan , J. Zhang , Y. Cai , C. Lan , B. Zhao , Y. Mei , Y. Zheng , E. Yang , X. Lu , X. Zhang , J. Tang , K. Yu , X. Zhang , Plant Commun. 2023, 4, 100682. 37691288 10.1016/j.xplc.2023.100682 PMC10721520

[41] C. H. Wellman , P. L. Osterloff , U. Mohiuddin , Nature 2003, 425, 282. 13679913 10.1038/nature01884

[42] D. Zhao , Z. Yao , J. Zhang , R. Zhang , Z. Mou , X. Zhang , Z. Li , X. Feng , S. Chen , R. J. Reiter , J. Pineal Res. 2021, 71, e12737. 33844336 10.1111/jpi.12737

[43] R. Vanholme , J. Ralph , T. Akiyama , F. Lu , J. R. Pazo , H. Kim , J. H. Christensen , B. Van Reusel , V. Storme , R. De Rycke , A. Rohde , K. Morreel , W. Boerjan , Plant J. 2010, 64, 885. 20822504 10.1111/j.1365-313X.2010.04353.x

[44] K. Yu , W. Yang , B. Zhao , L. Wang , P. Zhang , Y. Ouyang , Y. Chang , G. Chen , J. Zhang , S. Wang , X. Wang , P. Wang , W. Wang , J. A. Roberts , K. Jiang , L. A. J. Mur , X. Zhang , New Phytol. 2022, 235, 885. 35491444 10.1111/nph.18197

[45] S. D. Mansfield , H. Kim , F. C. Lu , J. Ralph , Nat. Protoc. 2012, 7, 1579. 22864199 10.1038/nprot.2012.064

[46] H. Kim , J. Ralph , Org. Biomol. Chem. 2010, 8, 576. 20090974 10.1039/b916070a PMC4070321

[47] A. M. Bolger , M. Lohse , B. Usadel , Bioinformatics 2014, 30, 2114. 24695404 10.1093/bioinformatics/btu170 PMC4103590

[48] D. Kim , J. M. Paggi , C. Park , C. Bennett , S. L. Salzberg , Nat. Biotechnol. 2019, 37, 907. 31375807 10.1038/s41587-019-0201-4 PMC7605509

[49] M. Pertea , G. M. Pertea , C. M. Antonescu , T.‐C. Chang , J. T. Mendell , S. L. Salzberg , Nat. Biotechnol. 2015, 33, 290. 25690850 10.1038/nbt.3122 PMC4643835

[50] P. Danecek , A. Auton , G. Abecasis , C. A. Albers , E. Banks , M. A. DePristo , R. E. Handsaker , G. Lunter , G. T. Marth , S. T. Sherry , G. McVean , R. Durbin , Bioinformatics 2011, 27, 2156.21653522

[51] P. J. Bradbury , Z. Zhang , D. E. Kroon , T. M. Casstevens , Y. Ramdoss , E. S. Buckler , Bioinformatics 2007, 23, 2633. 17586829 10.1093/bioinformatics/btm308

[52] N. Yang , Y. Lu , X. Yang , J. Huang , Y. Zhou , F. Ali , W. Wen , J. Liu , J. Li , J. Yan , PLoS Genet. 2014, 10, e1004573. 25211220 10.1371/journal.pgen.1004573 PMC4161304

[53] J. Yu , G. Pressoir , W. H. Briggs , I. Vroh Bi , M. Yamasaki , J. F. Doebley , M. D. McMullen , B. S. Gaut , D. M. Nielsen , J. B. Holland , S. Kresovich , E. S. Buckler , Nat. Genet. 2006, 38, 203. 16380716 10.1038/ng1702

[54] A. L. Price , N. J. Patterson , R. M. Plenge , M. E. Weinblatt , N. A. Shadick , D. Reich , Nat. Genet. 2006, 38, 904. 16862161 10.1038/ng1847

[55] S. Kumar , G. Stecher , M. Li , C. Knyaz , K. Tamura , Mol. Biol. Evol. 2018, 35, 1547. 29722887 10.1093/molbev/msy096 PMC5967553

[56] S. J. Clough , A. F. Bent , Plant J. 1998, 16, 735. 10069079 10.1046/j.1365-313x.1998.00343.x

