# Supplementary material for: Integrative Dissection of Lignin Composition in Tartary Buckwheat Seed Hulls for Enhanced Dehulling Efficiency
Source: Adv Sci (Weinh). 2024 Mar 23;11(20):2400916. doi: 10.1002/advs.202400916 (PMC11132045; doi:10.1002/advs.202400916)
Supplement: Supplementary file 1 — Supporting Information [file ADVS-11-2400916-s002.pdf]

ADVANCED SCIENCE
Open Access

Supporting Information

for *Adv. Sci.*, DOI 10.1002/advs.202400916

Integrative Dissection of Lignin Composition in Tartary Buckwheat Seed Hulls for Enhanced Dehulling Efficiency

Wenqi Yang, Haiyang Duan, Ke Yu, Siyu Hou, Yifan Kang, Xiao Wang, Jiongyu Hao, Longlong Liu, Yin Zhang, Laifu Luo, Yunjun Zhao, Junli Zhang, Chen Lan, Nan Wang, Xuehai Zhang, Jihua Tang, Qiao Zhao, Zhaoxia Sun* and Xuebin Zhang**

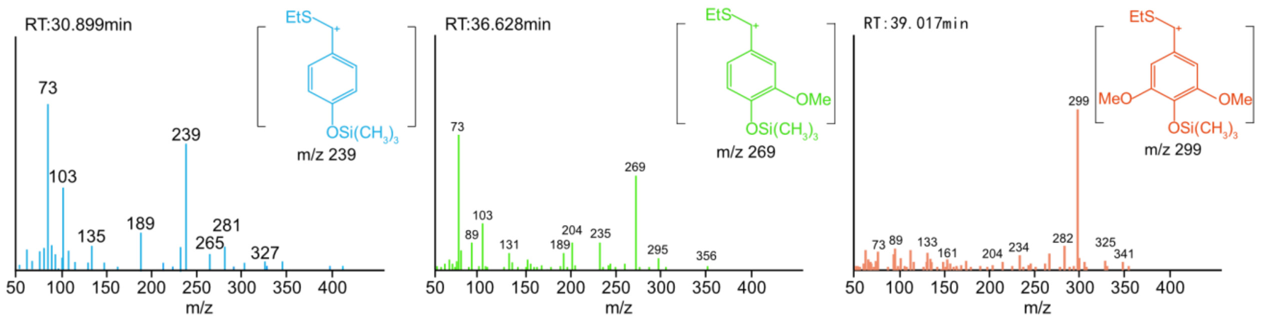

Figure S1. The representative mass spectra of H, G, S lignin unit in the thioacidolysis-released products of Tartary buckwheat cell wall from GC-MS.

The structures of characteristic base peak fragment ions are shown.

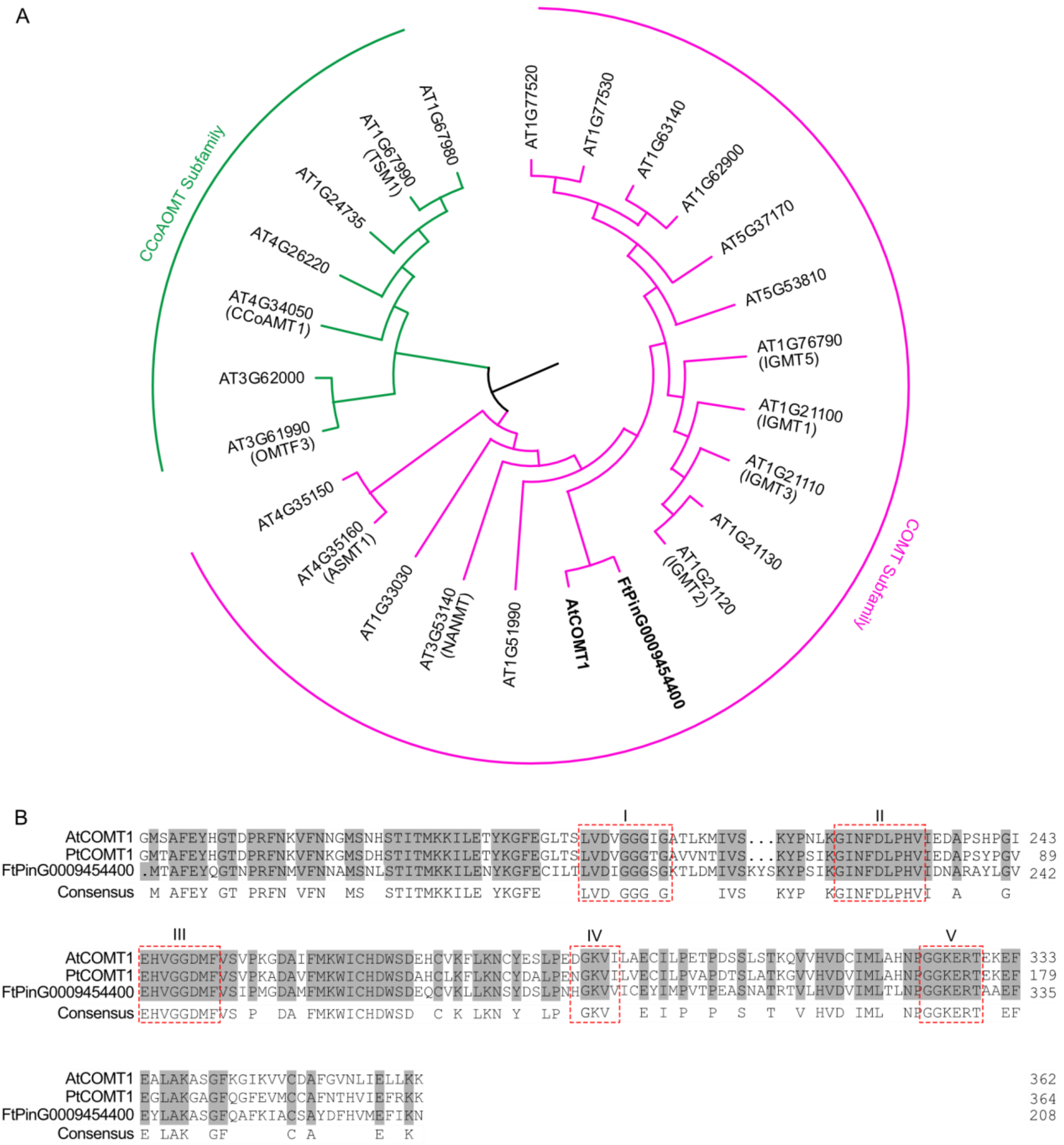

Figure S2. Phylogenetic analysis of FtPinG0009454400.

(A) Phylogenetic tree of representative OMT genes from *Arabidopsis* and FtPinG0009454400. The neighbor-joining method was used for clustering, and the tree was built by MEGA X. The cluster containing FtPinG0009454400 are bolded. (B) Protein sequence alignment of AtCOMT1, PtCOMT1 and FtPinG0009454400. Identical residues are high-lighted in grey, and the conservative domains are marked with red dashed line.

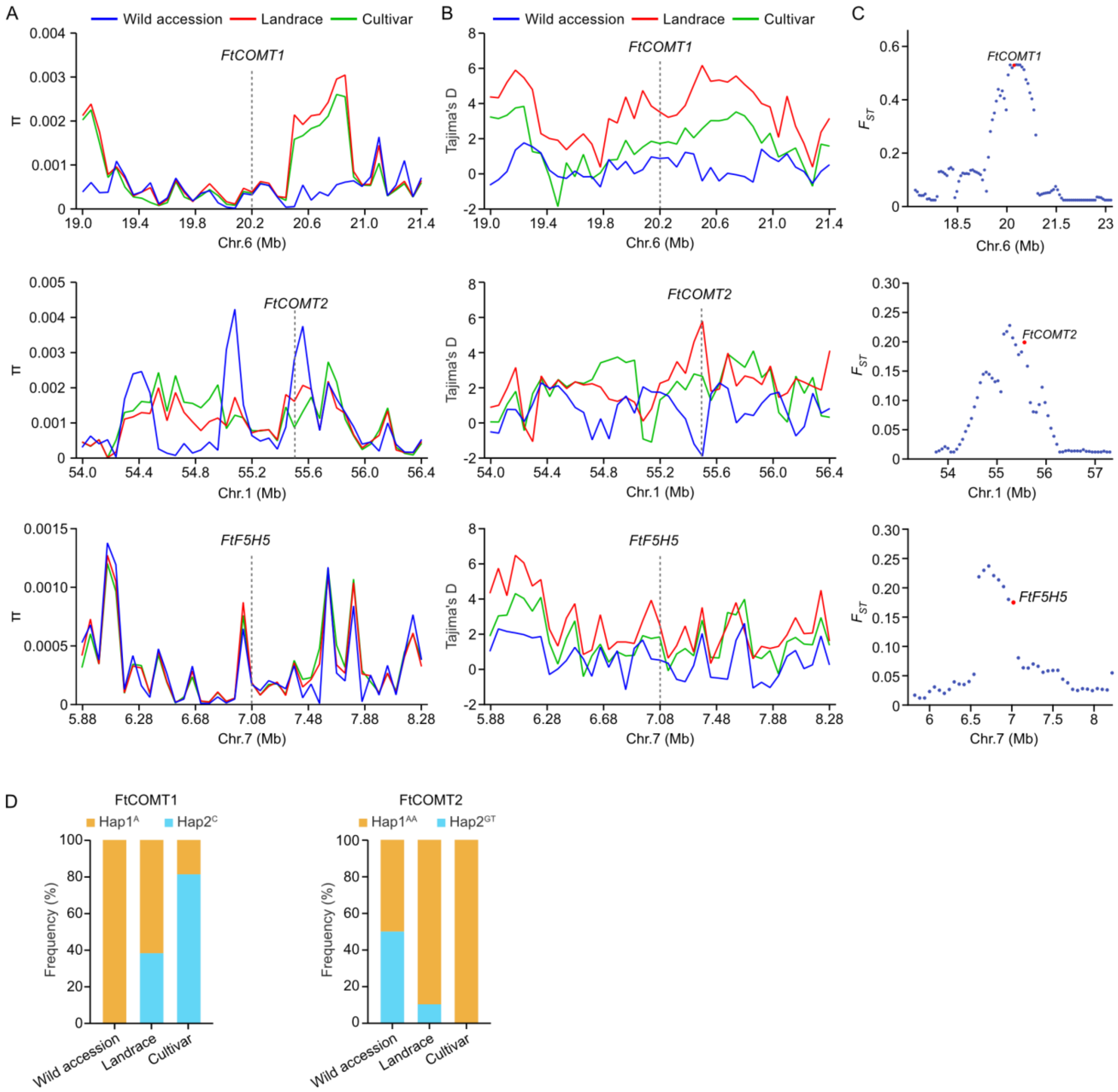

Figure S3 Validation of indicated selective sweep using Tajima's D and nucleotide diversity.

(A) Differential nucleotide diversity among three subpopulations at FtCOMT1, FtCOMT2 and FtF5H5 locus. (B) Tajima's D scores on the FtCOMT1, FtCOMT2 and FtF5H5 among three subpopulations. (C) 1 Mb flanking region signals (F_{ST} value) of indicated genes. (D) Frequencies of the two haplotypes of FtCOMT1 and FtCOMT2 in the wild accession, landrace and cultivar

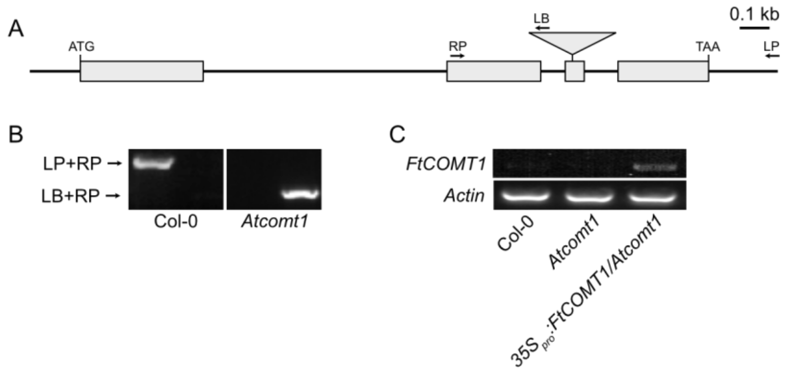

Figure S4. Identification of *Atcomt1* and transgenic line.

(A) Schematic representation of the genomic structure of the AtCOMT1 and T-DNA insertion site. (B) Genotyping of *Atcomt1*. (C) The expression of FtCOMT1 in indicated genotype.
